# Ecological interactions in *Cloudina* from the Ediacaran of Brazil: implications for the rise of animal biomineralization

**DOI:** 10.1038/s41598-017-05753-8

**Published:** 2017-07-14

**Authors:** Bruno Becker-Kerber, Mírian Liza Alves Forancelli Pacheco, Isaac Daniel Rudnitzki, Douglas Galante, Fabio Rodrigues, Juliana de Moraes Leme

**Affiliations:** 1 Programa de Pós-Graduação em Ecologia e Recursos Naturais, Universidade Federal de São Carlos, São Carlos (SP), Washington Luiz 325km, CEP 13565-905 Brazil; 2 Departamento de Biologia, Universidade Federal de São Carlos - campus Sorocaba, Rod. João Leme dos Santos km 110, CEP 18052-780 Sorocaba (SP), Brazil; 3 Departamento de Geologia, Universidade Federal de Ouro Preto, CEP 35400-000 Ouro Preto (MG), Brazil; 4 Brazilian Synchrotron Light Laboratory, Brazilian Center for Research in Energy and Materials, Av. Giuseppe Maximo Scolfaro, 10000, CEP 13083-100 Campinas, Brazil; 5 Departamento de Química, Instituto de Química, Universidade de São Paulo, Av. Prof. Lineu Prestes, 748, CEP 05508-000 São Paulo, Brazil; 6 Instituto de Geociências, Universidade de São Paulo, São Paulo, Rua do Lago, 562, Cidade Universitária, CEP 05508-080 Brazil

## Abstract

At the Ediacaran/Cambrian boundary, ecosystems witnessed an unparalleled biological innovation: the appearance of shelled animals. Here, we report new paleoecological and paleobiological data on *Cloudina*, which was one of the most abundant shelled animals at the end of the Ediacaran. We report the close association of *Cloudina* tubes with microbial mat textures as well as organic-rich material, syndepositional calcite and goethite cement between their flanges, thus reinforcing the awareness of metazoan/microorganism interactions at the end of the Ediacaran. The preservation of *in situ* tubes suggests a great plasticity of substrate utilization, with evidence of different life modes and avoidance behavior. Geochemical analysis revealed walls composed of two secondary laminae and organic sheets. Some walls presented boreholes that are here described as predation marks. Taken together, these data add further information regarding the structuring of shelled animal communities in marine ecosystems.

## Introduction

The Ediacaran Period (635–541 Ma) was marked by the record of enigmatic macroscopic organisms. This time interval embraces the first record of fossilized animals, including biomineralizers (e.g., *Namacalathus*, *Cloudina*)^1–3^, and vagile bilateria (e.g., *Kimberella*)^4^, as well as their innovative ecological interactions, such as macrophagous predation and competition^5, 6^. These evolutionary novelties led to the escalation and systematic organization of food webs, guilds and niches^7^ during the Cambrian radiation^8^. It was the dawn of animal life.

One of the key changes at the Ediacaran/Cambrian boundary was the disappearance of most macroscopic soft-bodied taxa. It is still a matter of discussion if this was a mass extinction event^9^. Nevertheless, recent data on Ediacaran soft-bodied organisms^1^ show that they were already declining in diversity, coinciding with the first appearance and spread of biomineralized metazoans (e.g., *Cloudina, Namacalathus*, *Namapoikia*) near the end of the Ediacaran – the ‘biotic replacement’ hypothesis.

Biomineralization among animals was one of the most impactful innovations in the history of life, and it affected sedimentological regimes^10^ and biogeochemical cycles^11^ and led to the complexification of benthic ecosystems^12–14^ and ecological interactions^5^.

Based on new data concerning modes of life, growth pattern/behavior, and the ecological interactions of the widespread genus *Cloudina*, we present new evidence that strengthens the role played by the new ecological opportunities (e.g., predation) that triggered unprecedented biotic changes (e.g., evolution of biomineralization among animals) near the Precambrian–Cambrian boundary.

## Results

### Association with microbialites

**Figure 1:**
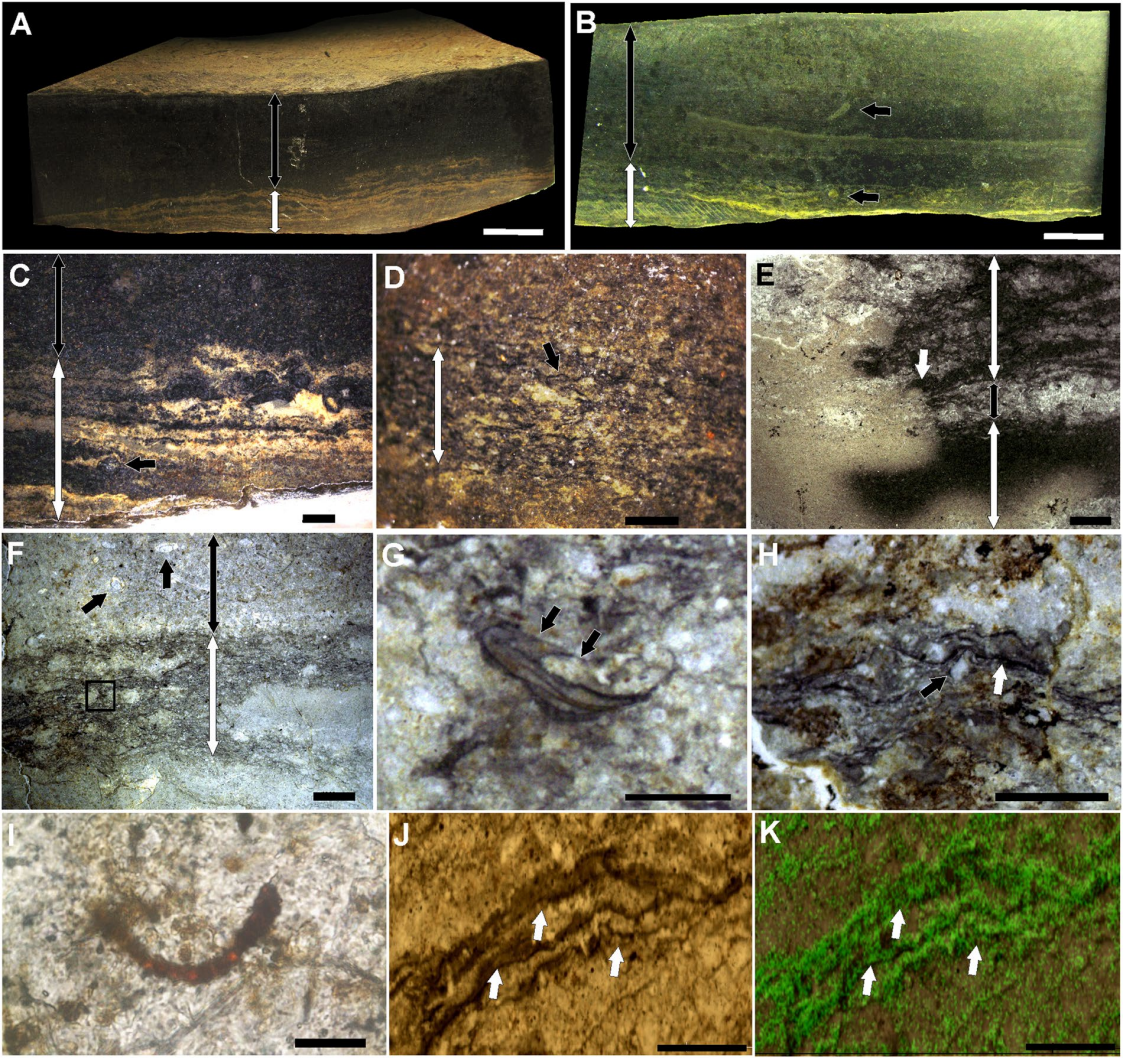
Biolaminites associated with *Cloudina*. (**A**) Polished hand sample showing the layer with microbial mat textures (white double-headed arrow) and the dark mudstone (black double-headed arrow). (**B**) Polished hand sample with a microbial mat layer (white double-headed arrow) and a dark mudstone layer (black double-headed arrow) and *Cloudina* oriented vertically (top black arrow) or inside microbial mats (bottom black arrow). (**C**) Polished hand sample with oxidized microbial mat textures (white double-headed arrow) associated with a *Cloudina* tube in cross-section (black arrow) and a mudstone layer above (black double-headed arrow). (**D**) Polished hand sample with microbial mat textures (white double-headed arrow) showing dark crenulated laminations (black arrow). (**E**) Thin section of microbial mat textures (white double-headed arrows) with an oxidation front (white arrow) and interbedded mudstones (black double-headed arrow). (**F**) Thin section demonstrating the presence of *Cloudina* inside crenulated laminations (black square and white double-headed arrow) at the base and associated with *Cloudina* (top black arrows) in a mudstone layer (black double-headed arrow). (**G**) Magnification of the black square in (**F**), showing a small individual of *Cloudina* inside the laminations and showing internal tubular structures (white arrow). (**H**) Thin section of the microbial mat textures showing dark lamination (white arrow) overlying occasional quartz grains (black arrow). (**I**) Microfossil of cyanobacteria. (**J**) Dark crenulated laminations (white arrows) of the microbialite associated with *Cloudina*. (**K**) Raman mapping of the region shown in (**J**) showing the presence of the “G” band of kerogen (green) mainly in the laminations (white arrows). Scale bars (**A,B**) 1 cm, (**C**,**E,F**) 2 mm, (**D**, **G,H**) 1 mm, (**I**) 20 µm (**J,K**) 0.2 mm.

Several (n = 291) autochthonous specimens of *Cloudina* were observed in association with microbialitic textures (biolaminites) in the dark limestones of the Tamengo Formation (Fig. 1; Supplementary Fig. S1). Petrographic analysis revealed crinkly lamination that trapped and bounded occasional quartz grains (Fig. 1H). The remains of *Cloudina* tubes are occasionally found inside the microbial mat laminations (Fig. 1B,C,F,G). Internal tubular structures were observed in one specimen found inside the microbial textures (Fig. 1G). Raman mapping indicated major concentrations of kerogen in the darker laminations of the microbial mat texture (Fig. 1K). The biolaminites are well preserved, with distinct margins and laminations (Fig. 1). In one polished hand sample, it was possible to observe that the macrostructure is laterally continuous and consists of an alternation of light and dark submillimetric laminations with low and crenulated synoptical relief (Fig. 1A–D); it occasionally shows oxidized laminations (Fig. 1C,E). The mesostructure is characterized by a plane laminated profile, crenulated laminations and moderate laminar heritage.

### Modes of occurrence

**Figure 2:**
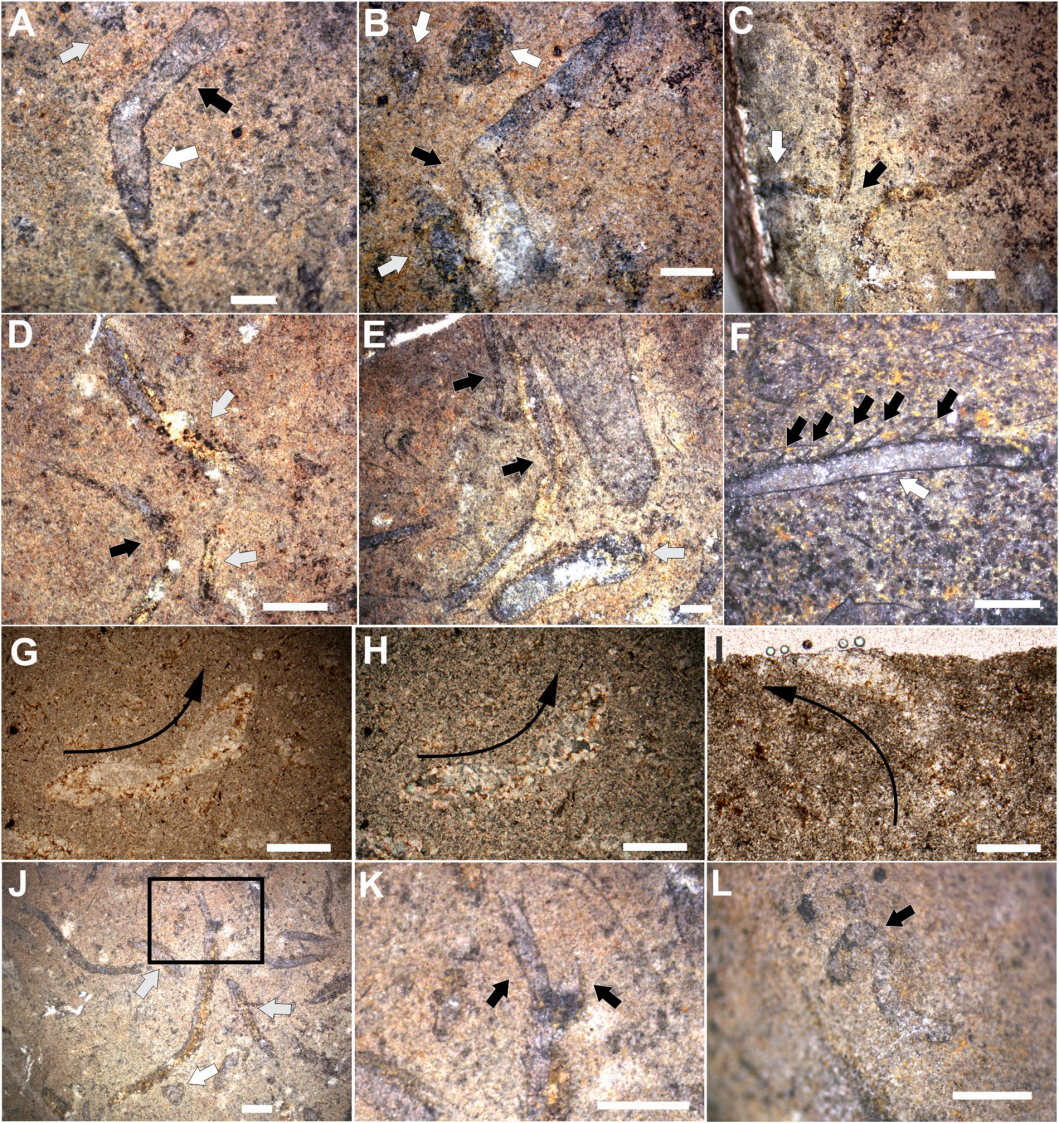
Horizontal to sub-horizontal life position of *Cloudina* specimens. (**A–E**) Drastic changes in the growth direction of a horizontally oriented specimen (black arrows) and a vertically oriented tube (white arrow). (**F**) Closed (white arrow) and open (black arrow) flanges preferentially in opposite sides of the tube. (**G,H**) Longitudinal section of *Cloudina* with horizontal and vertical orientation (black arrows showing the growth direction). Crossed nicols in (**H**). (**I**) Vertically oriented *Cloudina* (black arrow indicating growth direction). (**J**) Horizontally oriented tube with smaller tubes extending from the oral end. (**K**) Close view of (**J**) showing the daughter tubes. (**L**) Highly sinuous tube of *Cloudina*. Scale bars (**A**,**B**,**E,F,H**,**J–L**) 1 mm, (**C**,**D)** 2 mm, (**G**, **I**) 0.25 mm.

In samples with microbial textures, *Cloudina* tubes do not present a preferred orientation with respect to possible current sorting and are not fragmented or disarticulated. In fact, they present well-preserved flanges (Fig. 2C,F; Supplementary Fig. 2D–F). They exhibit high sinuosity (Fig. 2C,D,L; Supplementary Fig. 2C,D) and drastic changes in growth direction (Fig. 2A–E; Supplementary Fig. 2A,C), which can be spatially related to other tubes (Fig. 2A–E). One specimen has two smaller tubes extending from the oral end of a bigger individual (Fig. 2J,K). Despite some vertically oriented tubes (Figs 1B, 2B,C,G–I), most of the specimens (82%, n = 88) are preserved in the horizontal to oblique position, either inside the microbial textures (Fig. 1B,G) or inside the mudstone that overlies the microbial mats (Fig. 2). Some horizontal to oblique tubes are sinuously transecting the bedding plane (Supplementary Fig. 2B). There are also specimens that change from an oblique/vertical orientation to a horizontal position (Fig. 2A,C) and vice versa (Fig. 2G,H).

Only a few (n = 4) also presented closed and open flanges preferentially in opposite sides of the tube (bellow and above, respectively), but this is not a common feature (Fig. 2F). These fossils occur in an impure fine limestone, with microfacies defined as terrigenous mudstone. The matrix is represented by microspar and patches of sparry calcite that suggest a recrystallization of micrite. The terrigenous sediments consist of silt and fine sand grains, and they exclusively occur dispersed in the microspar matrix. The interior of the *Cloudina* tubes is filled mainly with sparry calcite and some microspar as cement obliteration. Microfossils were found in association with these autochthonous *Cloudina* (Fig. 1I).

### Calcite cement, goethite and organic-rich material inside the *Cloudina* flanges

**Figure 3:**
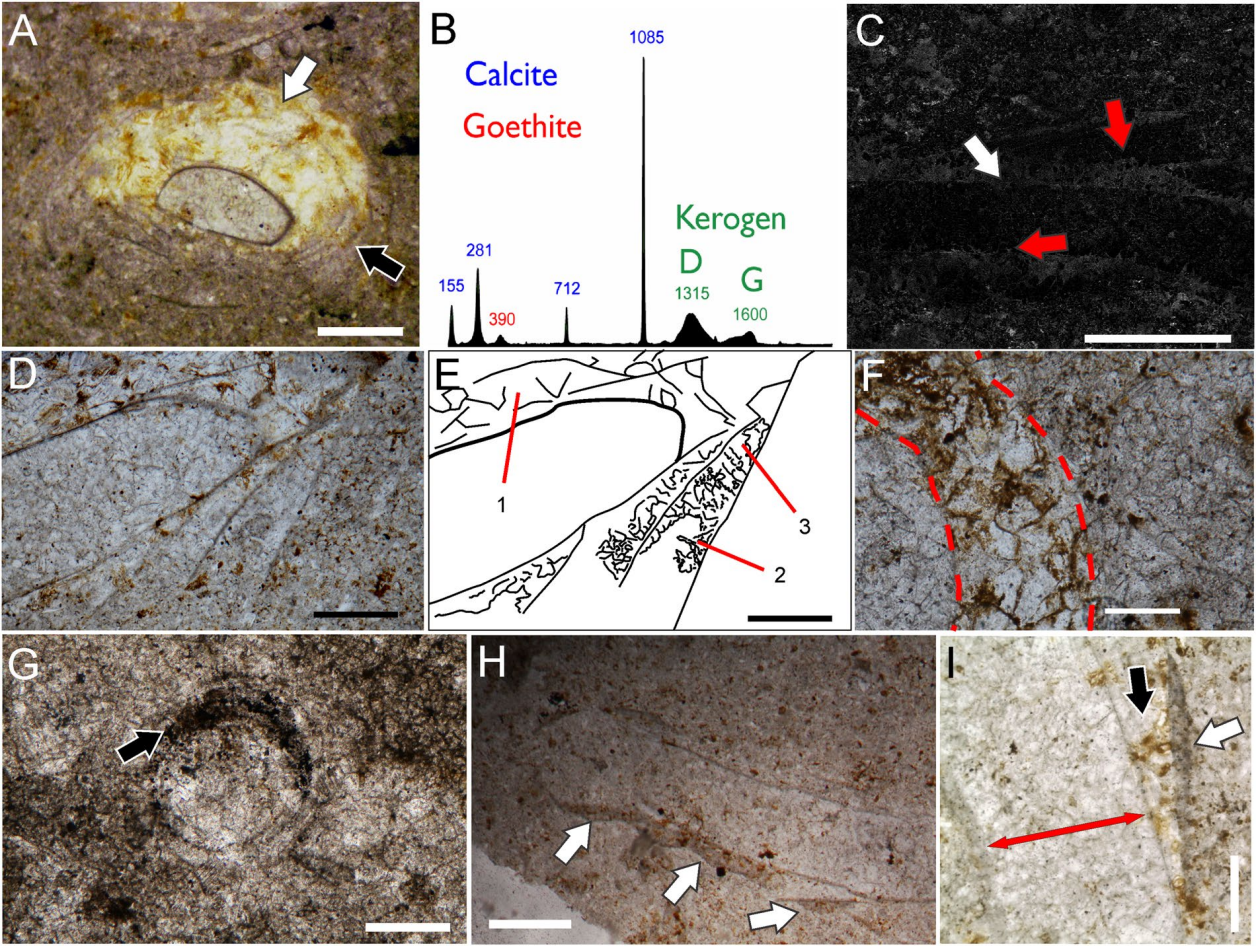
Syndepositional and diagenetic cement and organic-rich material inside the flanges of *Cloudina*. (**A**) Cross-section of a *Cloudina* tube showing patches of sparry calcite with intercrystalline goethite (white arrows) and patches of microspar and pseudospar (black arrows) between the wall layers. (**B**) Raman spectra of the region with cement showing bands of goethite, calcite and kerogen (**C**) SEM image of a tube in longitudinal section with sparry calcite in the innermost of the flanges (white arrow) and microspar towards the end of the flange (red arrow), the interior of the tube is also filled with microspar. Note the absence of siliciclastics inside the flanges (light areas of the host rock). (**D**) Longitudinal section of a tube, with sparry calcite and microspar-pseudospar cement between the walls. (**E**) Line drawing of (**D**) highlighting the areas with cement: (1) sparry calcite with intercrystalline goethite; (2) microspar; and (3) pseudospar. (**F**) Cross-section of a specimen showing the sparry calcite with intercrystalline goethite inside the flanges. (**G**) Cross-section of *Cloudina* with organic-rich material and opaque minerals (black arrow) inside the flanges. (**H**) Longitudinal section of *Cloudina* with preserved organic-rich material associated with the flanges (white arrows). (**I**) Longitudinal section of *Cloudina* showing patches of calcite cement (black arrow), and organic-rich material associated with the flanges (white arrows). Red double-headed arrow indicates the central canal of the tube. Scale bar (**A**,**H**) 0.5 mm, (**C**,**G**) 1 mm, (**D,E**) 0.25 mm, (**F**) 0.125 mm (**I**) 200 µm.

Calcite cement and intercrystalline goethite are ubiquitous inside the flanges of most autochthonous *Cloudina* funnels (83% of the specimens; Fig. 3A–F,I), whereas siliciclastic grains are absent (Fig. 3C–E; Supplementary Fig. S2). The calcite cement occurs as patches of microspar-pseudospar (Fig. 3A,C,D; Supplementary Fig. S2) and patches of sparry calcite with equidimensional mosaic texture (Figs 2H and 3A,C–F). Goethite occurs as intercrystalline inside the sparry calcite cement (Fig. 3A,D–F). In thin sections of the tubes, the filling framework shows two compositional zones of cement (Figs 2G–H and 3A,D–F): (i) the external zone (i.e., inside flanges), which consists of patches of microspar and pseudospar cement without goethite and patches of sparry calcite cement with intercrystalline goethite and pseudoframboids of pyrite; and (ii) the internal zone (tube interior), which is primarily composed of sparry calcite and microspar cement, absent the intracrystalline goethite. Organic-rich material was also associated with the flanges of *Cloudina* (Fig. 3G–I). Opaque minerals (i.e., iron oxides) and pseudoframboids of pyrite occur immersed in some of these organic remains (Fig. 3G,H). Transported specimens hosted in fossiliferous/intraclastic packstone show tubes with abraded flanges but with the external surface of the tube overlain by irregular molds of the cement filling (Supplementary Fig. S3).

### Holes in the shell

**Figure 4:**
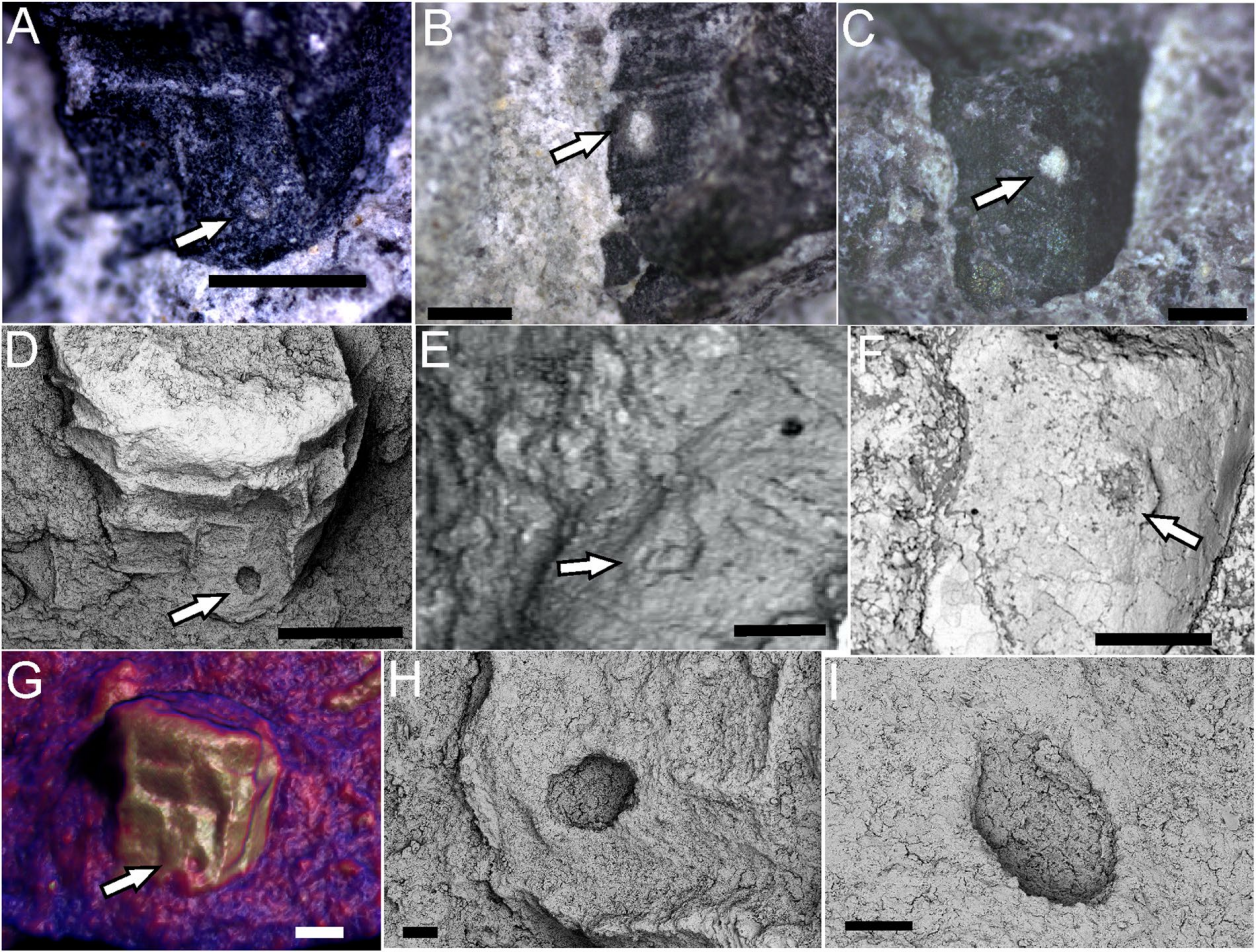
Possible predatory holes in *Cloudina*. (**A–C**) Stereomicroscope images of holes (white arrows) in *Cloudina* carapaces. (**D–F**) SEM images of the same specimens showing evidence of the circular outline of the holes. (**G**) Microtomographic image of the specimen in (**A**) and (**D**) showing the surficial hole in the shell (white arrow). (**H–I)** Zoom of the hole in (**D**) in different angles to show the perpendicular position with respect to the shell. Scale bar (**A**,**D**) 1 mm, (**B**,**C**) 0.5 mm, (**E**) 0.2 mm, (**F**) 0.5 mm, (**G**) 0.4 mm, (**H**,**I**), 0.1 mm.

Three specimens of *Cloudina* were noted to bear circular to ellipsoid holes in positions perpendicular to the tube (Fig. 4). The dimensions of these holes vary between 187 and 237 μm. Microtomographic analysis of these fossils demonstrated an area below the surficial hole that probably corresponds to the prolongation of the hole towards the interior of the tube (Supplementary Fig. S4).

### Microstructure and chemical composition

**Figure 5:**
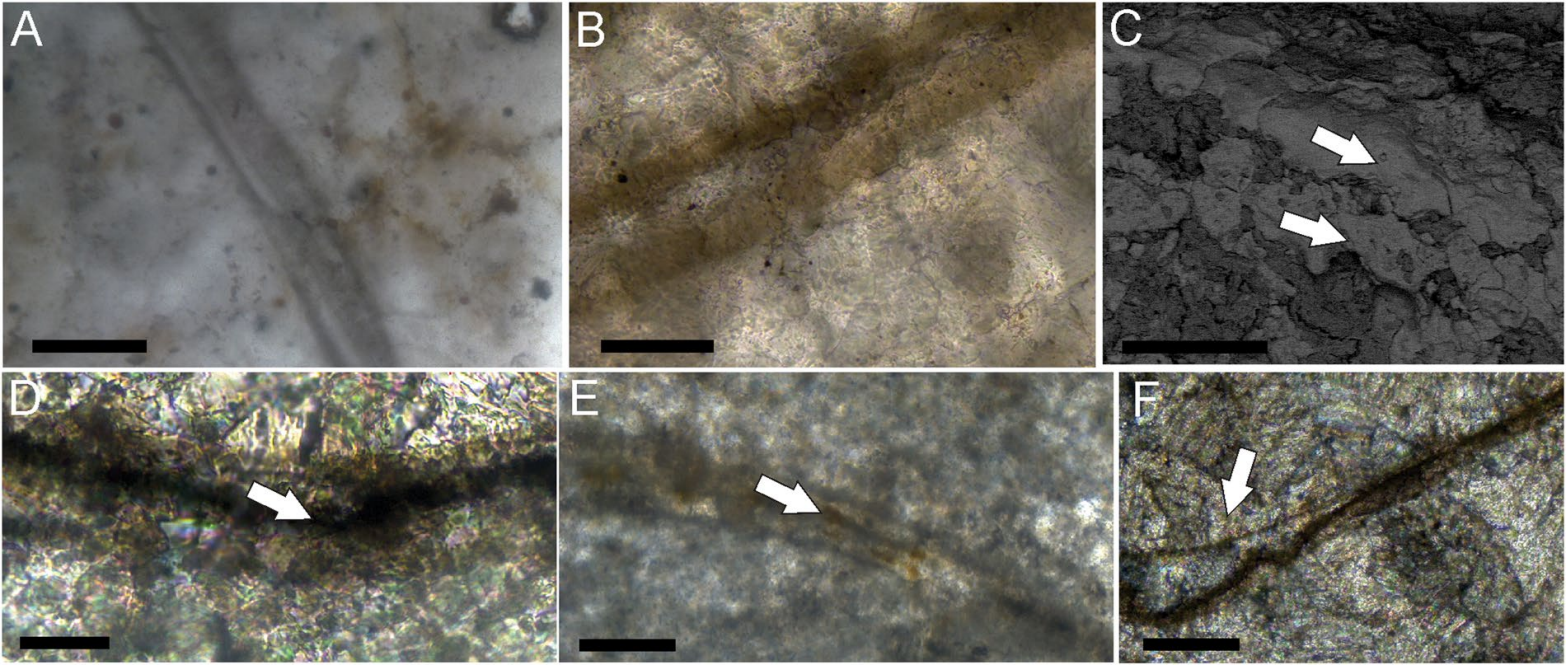
Two layered microstructure of *Cloudina* walls. (**A**,**B**) Thin sections of the shell showing the presence of two secondary laminae in cross-section. (**C**) Back-scattered electron (BSE) images of the two laminae in transverse section (arrows). (**D**,**E**) Dark interface between laminae in transverse section (arrows). (**F**) Deformed wall with a disconnected primary layer. Scale bars (**A**,**B**) 50 µm, (**C**) 40 µm, (**D**) 20 µm, (**E**,**F**) 100 µm.

**Figure 6:**
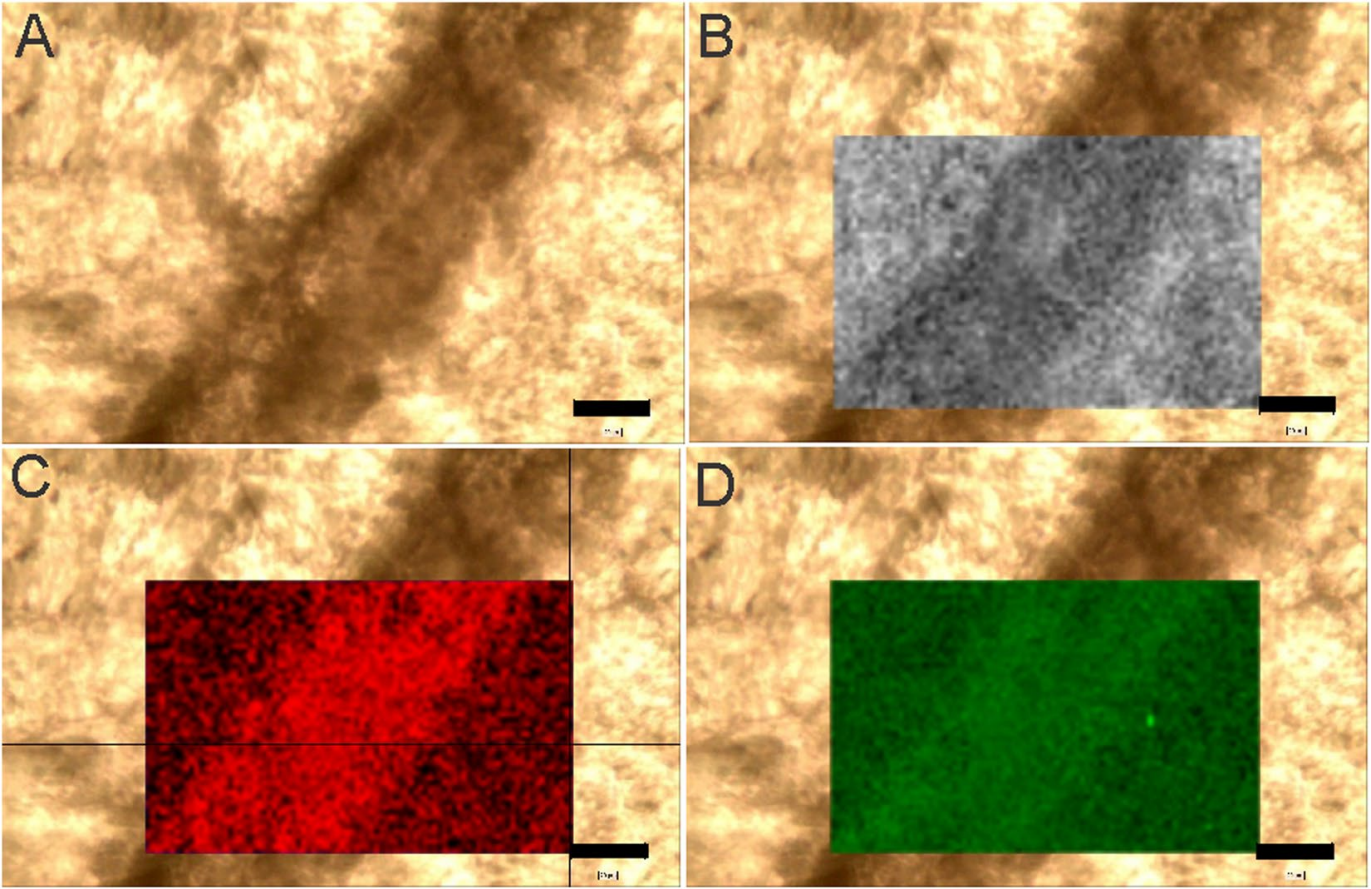
Raman mapping of *Cloudina* shell in transverse section. (**A**) Original image. (**B**) Mapping of calcite. (**C**) Mapping of the “D” band of kerogen. (**D**) Mapping of the “G” band of kerogen. Scale bar, 20 µm.

Thin sections and SEM of *Cloudina* revealed a shell that is primarily composed of two micrometric laminae (Fig. 5). Some deformed specimens demonstrated that these two laminae were composed of even smaller layers (Fig. 5F). Raman spectroscopy (Fig. 6; Supplementary Fig. S5) and energy-dispersive X-ray (EDS) mapping (Supplementary Fig. S6) of the shells showed the localized presence of kerogen and carbon, respectively. EDS point analysis revealed a low concentration of magnesium (Supplementary Table S1), and fitting of the Raman bands of the shells resulted in values of full width at half maximum (FWHM) similar to the calcite with low structural disorder due to low concentrations of Mg (Supplementary Table S2), as shown in previous studies^15^. Synchrotron-based X-ray fluorescence (SR-XRF) also presented the elemental constitution of the shell, specifically showing no difference in the Sr concentration between the fossil and the rock matrix (Supplementary Table S3).

## Discussion

The record of the most widespread and abundant Ediacaran shelled animals (e.g., *Cloudina*, *Namacalathus*, *Namapoikia*) has been found associated with different kinds of microbialites. Examples have been described in stromatolitic biostromes in the Byng Formation^16^, bioherms of the Ara Formation^17^, stromatolitic and thrombolitic biostroms in the Nama Group^18^, thrombolites in the Taguatiya Guazu Formation^19^ and microbial biolaminites of the Dengying Formation^20^.

Microfossils of cyanobacteria preserved together with autochthonous *Cloudina* (Fig. 2L) reinforce metazoan-microbialite interactions. The observation of *Cloudina* within and associated with biolaminites (Fig. 1) in this work extends the paleogeographical occurrence of metazoan/microbialite associations. It reinforces that *Cloudina* had a great ecological flexibility and was able to colonize different forms of microbial substrates^20^.

It is worthwhile to consider the impact that photosynthetic microbial communities could have had in the evolution of hard parts. During day periods, extant photosynthetic microbial mats can increase the local concentration of oxygen and exceed the atmospheric levels^21^; for this reason photosynthetic matgrounds may have played a role in the early evolution of mobile bilateria during the Ediacaran^22^. Regarding the evolution of biomineralization in animals, an oxygen oasis can also constitute a suitable habitat for the evolution of the metabolically costly skeletons of the metazoans, thereby assigning another ecological role to microbial mats in Ediacaran ecosystems^22–24^.

The *Cloudina* association with microbial biofilms from Tamengo was even more intimate than ever reported for animals with shells in the Ediacaran. The framework of flange void filling and evidence of cement in transported specimens (Fig. 3A–F; Supplementary Figs S2 and S3) reveal pertinent clues regarding the obliteration evolution. The absence of siliciclastic material inside the flanges demonstrates that a mechanical barrier was present before the burial stage and prevented the input of sediment (Fig. 3C; Supplementary Fig. 4). The organic-rich material and opaque minerals found internally to the flanges (Fig. 3G,H) and, in some cases, externally (Fig. 3H,I) may represent an original organic mass that acted as a mechanical impediment or the remains of microbial communities that developed in the space between the layers of the wall. Furthermore, the pseudoframboids of pyrite and opaque minerals (e.g., iron oxides) found immersed within these organic remains suggest the anaerobic decomposition of this original organic mass. Additionally, the presence of transported tubes with irregular coverings of calcite cement, abraded flanges, and sediments in the central canal of the transported tubes (Supplementary Fig. S3) strongly suggests that, at least, some calcite cement is syndepositional or early diagenetic. Similar conditions were observed for the *Cloudina* remains from the Nama Group and the Membrillar olistostrome of Spain^25, 26^. Therefore, it is likely that early marine cements also played a role in the prevention of sediment infilling.

The microspar cement (Fig. 3A,C–F; Supplementary Fig. 4) likely represents the recrystallization of autochthonous micrite and can represent syndepositional cement. Taken together, (1) the precipitation of autochthonous micrite, (2) the presence of organic remains (Fig. 3G–I) between *Cloudina* walls and (3) a close association with microbial mats were all interpreted as products of microbial metabolism^27, 28^ and the calcite cement between the walls of *Cloudina*. This bears similarities to the extant synsedimentary lithification of modern marine polychaetes due to the precipitation of carbonate by symbiotic bacteria^29^. This interpretation also has implications for other enigmatic structures that were found to be associated with *Cloudina*, such as the meniscate forms in the reefs of Namibia^13^.

**Figure 7:**
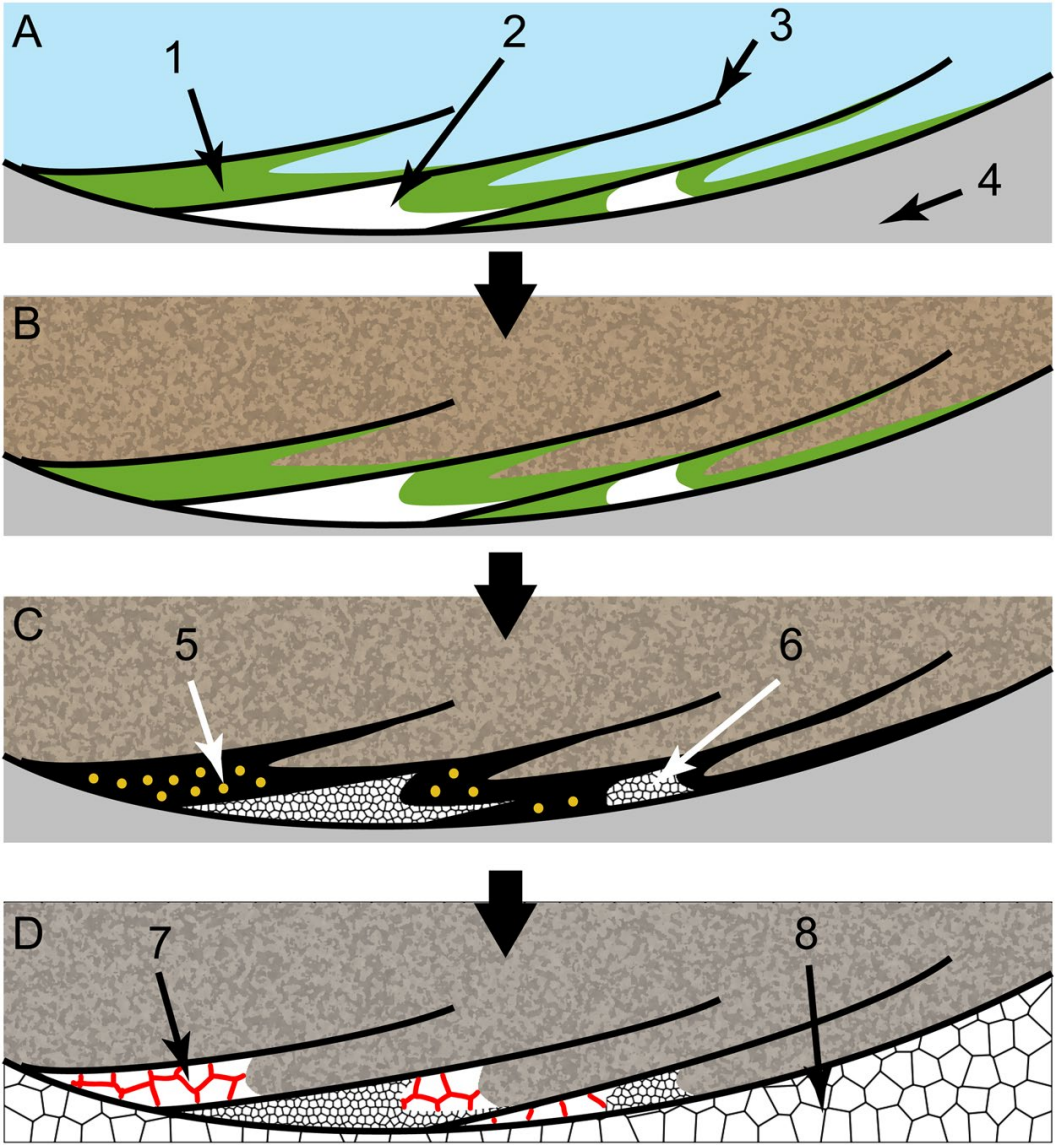
Model for the origin of calcite cement, organic-rich material and intercrystalline goethite between the walls of *Cloudina*. (**A**) Longitudinal section of the flanges of a *Cloudina* living on the water/sediment interface and/or microbial mats. 1: microbial mass; 2: early marine micrite; 3: flange; 4: central canal of the tube. (**B**) Burial and prevention of sediment infilling inside the flanges. (**C**) Early diagenesis with anaerobic decomposition (5) and recrystallization of micrite to microspar and pseudospar (6). (**D**) Posterior diagenetic processes of cement obliteration with precipitation of sparry calcite encasing goethite into intercrystalline spaces (7) inside the flanges and precipitation of sparry calcite in the central canal of the tube (8).

Enclosed organic matter or irregular micrite fillings between the wall also left behind empty pore spaces that were later cemented by sparry calcite and intercrystalline goethite (Figs 2G,H, 3A,C–F and 7). The preferential occurrence of intercrystalline goethite in the sparry calcite cements of the flanges suggests that this iron oxide resulted from the oxidation of iron sulphates (e.g. pyrite) that originated from anaerobic decomposition of the organic mass that was retained inside the flanges (Fig. 7). Another possibility for the intercrystalline goethite is the infiltration of iron oxides in permoporous rocks. However, the presence of associated pseudoframboids of pyrite in organic remains and in the cements between the flanges suggests that an original pyritic material could have been the precursor of the intercrystalline goethite.

This model suggests an intimate association between *Cloudina* and biofilms (Fig. 7). Two hypotheses are suggested from this interaction: i) microbial biofilms proliferated in the decaying carcass of *Cloudina* prior to burial of the organisms; and ii) microorganisms were associated with living *Cloudina*, establishing an ecological relationship (e.g., amensalism, neutralism, commensalism or mutualism). The preferred localization of the organic-rich material and cement inside the flanges sustained the later (Fig. 3G–I). In some specimens (e.g., Fig. 3A), the presence of goethite and calcite cement preferentially on one side of the surface of the tube may be related to the space available for the development of biofilms, thus reinforcing a prostrate life mode, perhaps even a partially buried one. Alternatively, these structures can be interpreted as geopetal features, but this is refuted by the lack of a preferential vertical position with respect to stratigraphy. In addition, geopetal structures would not explain the mechanical barrier present before burial (Fig. 3C–E), the occurrence of organic matter remains or transported tubes with ghosts of the funnel-in-funnel structure created by early cement and abrasion of the walls (Fig. 3G–I; Supplementary Fig. S3).

Independent of the type of interaction, the deposition of early cement inside the flanges of *Cloudina* likely played a role in the mechanical rigidity of the shell, as previously proposed^25, 26^, and possibly also influenced the construction of the *Cloudina* reefs from Namibia.

**Figure 8:**
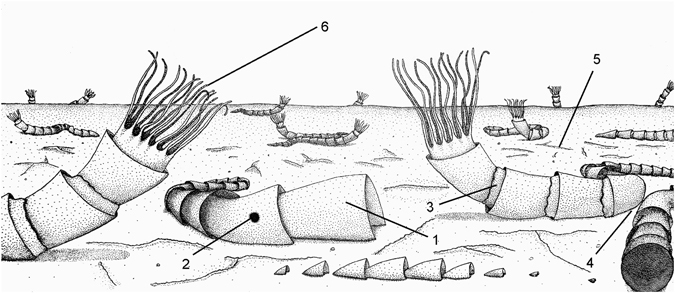
Reconstruction of the living *Cloudina*. 1: funnel segment of *Cloudina* tubes; 2: boring holes made by macrophagous predators; 3: biofilms that developed in association with the *Cloudina* external surface; 4: changes in growing direction related to space competition; 5: microbial mats; 6: conjectural tentacle-like structures.

In addition to ecological flexibility (i.e., occurrences in different types of microbial substrates)^16–20^ and related ecological interactions with microorganisms (Figs 1–3 and 7), *Cloudina* evolved a plasticity of substrate utilization through different ways of growth and life modes^14, 20^. A horizontal life position is the main growth mode in the reef-buildups of *Cloudina* from Namibia^14^, although they occasionally present erect growing behavior in other localities^19, 20^. For the limestones of the Tamengo Formation, a horizontal/oblique with occasional vertical life mode is proposed (Fig. 8). Vertically oriented tubes strongly support the *in situ* preservation of the fossils (Fig. 2B,C,G–I). Because currents and transportation would rearrange the fossils parallel to the bedding plane, the presence of specimens that change from an oblique/vertical to horizontal orientation (Fig. 2A,C) and vice versa (Fig. 2G,H), and specimens that sinuously transect the bedding plane further reinforces that these fossils are *in situ*. Furthermore, the high degree of preservation of delicate structures corroborated that no transport occurred (Supplementary Fig. 2E,F). Therefore, the fossils of this autochthonous assemblage are interpreted as having a horizontal to oblique life mode with occasional vertical growth. Tubes that exhibit high sinuosity (Fig. 2L), similar to the “cobra-like” morphology^20^, and drastic changes in growth direction spatially related to other tubes of *Cloudina* also highlight a horizontal life mode (Figs 2A–E and 8). Furthermore, the preferential closed flanges in one side of some tubes (Fig. 2F) could have been a response to a horizontal life mode for some individuals in which the organism could increase the surface area in contact with the sediment/matground. Otherwise, this kind of tube construction and the drastic changes in growth direction would be unstable in a straight vertical orientation of the tube.

The horizontal to oblique mode of life with occasional vertical growth (Fig. 8) is consistent with previous interpretations of *Cloudina* ecology from other localities^13, 19, 20^ and reinforces the plasticity in substrate utilization for this gregarious filter-feeding metazoan. In addition, changes in growth direction that are spatially related to other tubes are unlikely to have been randomly generated by currents (Fig 2A–E). However, this evidence can reflect intraspecific competition for space in a mechanism of avoidance, similar to the stand-off strategy common in some (recent and fossil) clonal organisms^30^. Intraspecific competition in recent communities is related to crowding and resource-weighted density, and these factors were probably involved in *Cloudina* and other paleocommunities. In fact, the apparent absence of competitive behavior in the *Cloudina* reefs of Namibia suggests that the two environments may have been different in their carrying capacity, including the fluctuations in nutrient levels. This capacity of *Cloudina* to change its growing direction in the presence of possible space competitors shows a greater degree of sensorial capacity than previous thought for macroscopic life at the Ediacaran/Cambrian transition.

Although the ecological context of the end of the Ediacaran may have been favorable for the evolution of biomineralization in animals, the advent of predators is still a strong possible trigger for the appearance of hard parts^7, 31, 32^. The observation of holes in *Cloudina* from the Tamengo Formation (see Supplementary Text 2 for the diagnostic criteria of predatory holes) strengthens the relation between the first shells and macrophagous feeding styles among metazoans (Figs 4 and 8). In addition, the gregarious habit of *Cloudina* shown on the reefs of the Nama Group reinforces the possibility of increasing levels of predation during that period^13^.

Hence, it seems that these first shelled metazoans possessed more complex biomineralized parts than previously thought. Here, we verified a double laminated wall in *Cloudina* from the Tamengo Formation. Considering previous interpretations of *Cloudina* walls as multiple primary layers (each one with the size of calcite crystals) forming a secondary lamina^2^, our results suggest that the wall of *Cloudina* may have been composed of at least two secondary laminae (Fig. 5). It is likely that the degree of preservation of the Tamengo material prevented the observation of the primary layers. However, some deformed specimens with disconnected small lamina may correspond to these primary layers in the microstructure of *Cloudina*. The carapace also had high organic content evidence in our samples, as shown by Raman point spectra and mapping and EDS mapping (Fig. 6; Supplementary Figs 7 and 8). The darker interface between the laminae (Fig. 5D–F) may even represent original organic sheets in the skeleton of *Cloudina*. The interaction between biominerals and organic sheets (double laminae and organic sheets) could have had a great effect on the mechanical resistance of the tube of *Cloudina*, similar to what is found in recent serpulids^33^.

Contrary to the microstructural characteristics of *Cloudina*, the original composition of the mineral is less defined^34, 35^. We detected low concentrations of Mg (Supplementary Tables S1 and S2) in the *Cloudina* shells from Tamengo. Nonetheless, this does not necessarily imply that the *Cloudina* shells had low-Mg calcite mineralogy because diagenetic stabilization can cause a significant loss of Mg^34–36^. However, another approach to looking for the original mineralogy of a fossil is to analyze the values of Sr: a stronger concentration of Sr (approximately >1000 ppm) in the fossil compared to the rock indicates original aragonite composition^37^. Considering that the concentration of Sr tends to be stable during the diagenetic alteration of aragonite to calcite^38^, the similar values of Sr between *Cloudina* and the rock suggest, at least, a calcitic nature for *Cloudina*, corroborating previous interpretations that were based on associated cements, microdolomite inclusions and preferred dolomitization^2^. This is important information for modeling the first phase of biomineralization in the history of animal life in relation to extrinsic (e.g., aragonite/dolomite, aragonite and calcite seas) and intrinsic (e.g., ecology) factors^7, 37–39^. Knowing the original mineralogy of fossil skeletons is of key importance to understanding the evolutionary patterns through geological time^7^.

It has long been argued that ecological interactions may have played an important role in the diversification of animals in the beginning of the Cambrian, leading to ecological escalation and co-evolution of predators and prey. The ecological and mineralogical characteristics of *Cloudina* such as space utilization, close association with microorganisms and a complex shell structure may have helped with its establishment in environments with extrinsic and intrinsic stress factors, such as fluctuating levels of oxygen^24^ and hydrogen sulphide^40^ and increasing rates of macrophagous predation^5, 6^, this study. Finally, the associations of *Cloudina* and microbial mats in the Tamengo Formation corroborate this paleoenvironmental setting as a possible scenario for the origin of the first biomineralizers. It is possible that shallow waters dominated by microbialites produced favorable conditions for the evolution of biomineralization.

## Methods

### Provenance of the fossils

The *Cloudina* fossils studied here come from the carbonate sediments of the Tamengo Formation (Supplementary Text 1 and Supplementary Fig. S1), a late Ediacaran unit (543 ± 3 Ma) of the Corumbá Group^41^ (Paraguay Belt, Mato Grosso do Sul, Brazil). The main localities include the Corcal and Laginha quarries and Sobramil harbor in Corumbá city and one outcrop in Ladário city (Supplementary Text 1 and Supplementary Fig. S1). The fossils are deposited in the scientific collection of Paleontology of the Geosciences Institute, University of São Paulo.

Digital images for all specimens were captured under bright-field illustration using an AxioCam ICc 3 digital camera mounted to a Stereoscope Zeiss Stemi 2000-C. Petrographic analyses were performed with a Zeiss Axioplan 2 microscope, and the images were obtained with an MC 170 HD camera, and processed by Leica LAS 4.4 software. All images were later processed in Adobe Illustrator.

### Raman configuration

The equipment utilized was a Renishaw InVia microRaman with 633 nm and 785 nm lasers, 17 mW total power (attenuated to 0.1%, 0.5%, 1%, 5% e 10%), and variable exposure and accumulation time. The spectra were processed using WiRE 4.1 and OriginPro8 software. The Raman mapping data were obtained using the streamline method.

### X-ray Fluorescence (SR-XRX)

The measurements were performed at the XRF beamline of the Brazilian Synchrotron Light Laboratory (LNLS)^42^ in microbeam mode, using polychromatic excitation, filtered with Fe and Al foils, and a KB focusing system to achieve a beam size of approximately 12 × 25 μm on the sample. Mappings were made with steps of 50 μm, on matrices of 70 × 70 points, covering 3.5 × 3.5 mm. The accumulation time was of 1 s per point. All data were treated using PyMCA software^43^ to calculate the absolute values of concentration for each element using a NIST 612 trace-elements standard. Control spectra were collected on the glass slide, on the glue and on the rock matrix to ensure that real data from the fossil could be discriminated from eventual contaminants.

### Microtomography

The microtomographic analyses were realized using a Skyscan 1176 High-Resolution *In-Vivo* Micro-CT of the Microtomographic Laboratory of Biosciences Institute, São Paulo University (USP), with a maximum resolution of 9 µm, a voltage source of 90 kV and a current source of 278 uA.

## Electronic supplementary material

Supplementary Information

Supplementary Table S1

Supplementary Table S2

Supplementary Table S3
