# Supplementary material for: Ecological interactions in Cloudina from the Ediacaran of Brazil: implications for the rise of animal biomineralization
Source: Sci Rep. 2017 Jul 14;7:5482. doi: 10.1038/s41598-017-05753-8 (PMC5511220; doi:10.1038/s41598-017-05753-8)
Supplement: Supplementary file 1 — Supplementary Information [file 41598_2017_5753_MOESM1_ESM.pdf]

Supplementary Information for

Ecological interactions in *Cloudina* from the Ediacaran of Brazil: implications for the rise of animal biomineralization

Bruno Becker Kerber[1*], Mírian Liza Alves Forancelli Pacheco[2], Isaac Daniel Rudnitzki[3], Douglas Galante[4], Fabio Rodrigues[5], Juliana de Moraes Leme[6]

[1]. Programa de Pós-Graduação em Ecologia e Recursos Naturais, Universidade Federal de São Carlos, São Carlos (SP), Washington Luiz 325 km, CEP 13565-905, Brazil; e-mail: bruno.becker92@gmail.com

[*]Corresponding author

[2]. Departamento de Biologia, Universidade Federal de São Carlos - campus Sorocaba, Rod. João Leme dos Santos km 110, CEP 18052-780, Sorocaba (SP), Brazil.; e-mail: forancelli.ufscar@gmail.com

[3]. Departamento de Geologia, Universidade Federal de Ouro Preto, CEP 35400-000, Ouro Preto (MG), Brazil; e-mail: idrgeo@gmail.com

[4]. Brazilian Synchrotron Light Laboratory, Brazilian Center for Research in Energy and Materials, Av. Giuseppe Maximo Scolfaro, 10000, CEP 13083-100, Campinas, Brazil.

[5]. Departamento de Química, Instituto de Química, Universidade de São Paulo, Av. Prof. Lineu Prestes, 748, CEP 05508-000, São Paulo, Brazil.

[6]. Instituto de Geociências, Universidade de São Paulo, São Paulo, Rua do Lago, 562, Cidade Universitária, CEP 05508-080, Brazil; e-mail: leme@usp.br

Supplementary Text 1. Geological setting

Corumbá Group crops out on the Southern Paraguay Belt at Mato Grosso do Sul state (Southwest of Brazil). It is inserted in the geological context of the Rio Apa Block and probably was developed in a rift-to-drift scenario (Alvarenga *et al*. 2000) as a mixed carbonate-siliciclastic plataform (Boggiani et al. 2010). Corumbá Group overlies conformably the Puga Formation, which consists in glaciogenic diamictites related to the Marinoan glaciation event at 635 Ma (Alvarenga & Trompette 1993, Boggiani et al. 2010). Isotopic studies performed on the Corumbá Group found $\delta^{13}C$ and $^{87}Sr/^{86}Sr$ curves that are comparable with other late Ediacaran units, as Nama and Oman (Grotzinger *et al*. 1995; Amthor *et al*. 2003; Boggiani *et al*. 2010). Additionally, the presence of the index fossil *Cloudina* and other Ediacaran fossils (*Corumbella*, leiosphaers and vendotaenids), associated to a volcanic tuff interlayer with 543±3 Ma age (U-Pb SHRIMP zircon) at the upper part of the Corumbá Group, Tamengo Formation, corroborates the late Ediacaran age for this geologic unit (Gaucher *et al*. 2003; Babinski *et al*. 2008; Alvarenga *et al*. 2009; Boggiani *et al*. 2010).

With a thickness of approximately 600 m (Gaucher *et al*. 2003), the Corumbá Group is composed of sandstone and siltstone of the Cerradinho Formation, followed by dolostone, microbialite and phosphorites of the Bocaina Formation (Supplementary Figure S1). The Bocaina Formation is overlaid by the shale, limestone and marl beds of the Tamengo Formation. Finally, the Corumbá Group ends with the black shale beds of the Guaicurus Formation (Almeida 1965, 1984; Boggiani 1998; Trompette *et al*. 1998; Alvarenga *et al*. 2000; Gaucher *et al*. 2003; Boggiani *et al*. 2010). The abundant remains of metazoan fossils *Cloudina* and *Corumbella*, microfossils (*Myxococcoides* sp., *Bavlinella faveolata*) and

vendotaenids (*Vendotaenia antique*, *Eoholynia corumbensis*), characterize Tamengo Formation (Zaine and Farichild 1985, 1987; Zaine 1991; Gaucher *et al*. 2003; Pacheco *et al*. 2011; Fairchild *et al*. 2012). More specifically, Tamengo Formation comprises fine limestone with siliciclastic intercalation and intraclastic breccia at the base, changing towards the top to shales, bioclastic grainstones and rudstones with hummocky cross-stratification and fine limestone (Supplementary Figure S1). This sequence is interpreted as a shallow marine environment that sometimes displays more deep water character (Spangenberg *et al*. 2013). The presence of grainstones and rudstones together with hummocky cross-stratification suggests intermittently high-energy events in an environment usually with low-energy (Spangenberg *et al*. 2013).

The analyzed fossil samples were previously collected in Tamengo Formation (see Zaine 1991; Meira 2011) and are deposited at the Paleontological Collection of the Institute of Geosciences (University of São Paulo – USP). Some thin sections (GP/1E-89; 8/19) showing microbial mats are from limestones from the rhytmite facies (limestone-marls) of Laginha quarry, Corumbá region, supported by a profile with ~70 m thick. The Laginha section was previously described by lithological features and the depositional environment interpretation followed their description (for more detail see Boggiani et al. 2010). The basal section consists of polymictic carbonate breccias interpreted as slope deposits. It is overlaid by massive fine limestone (mudstone and wackestone) and oolitic/bioclastic grainstones, followed by a thick package of rhytmite of limestone-marls representing deposits of deep water to transitional conditions. The uppermost section consists of grainstone with ooids and bioclast defined as shoal shallow water. *Cloudina* associated with microbial textures were found in mudstones of one outcrop in Ladário city, defined by a profile of ~13 m, composed mainly by mudstone with intercalated levels of 1-2 m thick

pelites (Supplementary Figure 1). Lithologically correlated with these, thin sections and hand-samples with fine limestone also presented autochthonous *Cloudina* (e.g. GP/L1E-41-42; GP/1E-6218).

Supplementary Text 2. Diagnostic characteristics of boring holes

Boring holes in *Cloudina* were already reported for phosphatized specimens from Dengying, China (Bengston and Zhao, 1992; Hua *et al*., 2003) and two calcified individuals from Nama Group (Brain, 2001). These structures were considered to represent the oldest evidences of the fossil record for predatory activity in animals. The presence of holes in fossils of *Cloudina* from Tamengo Formation marks the third occurrence and geographically expands the possible evidences of predation near the Ediacaran/Cambrian boundary.

Circular holes in marine invertebrates, when of predatory origin (i.e. boreholes or drillholes), constitute an important tool for measuring ecological interactions in the fossil record. However, differentiating holes produced by predators from that originated by other mechanisms is not so simple (Kowalewski, 2002). Among the criteria utilized for recognition of boreholes are: [1] one hole for prey; [2] perpendicular position in relation to the shell; [3] sharp edges; [4] selectivity for prey size; [5] selectivity for location of the hole; [6] selectivity for prey species; [7] incomplete holes (Kowalewski, 2002; Harper, 2003).

The holes analyzed here meet the criteria 1 to 3, and possibly the criteria 6. Until now, there are no reports of holes on *Corumbella*, and in the same way for *Cloudina* and *Sinotubullites* of Dengying Formation, this could represent selectivity for prey species. While the low

sample size of bored specimens from Tamengo Formation precludes statistical analysis to test location and/or size selectivity (criteria 4 and 5), these conditions were observed in Dengying Formation (Bengtson and Zhao, 1992; Hua *et al*., 2003), together with the presence of incomplete holes (criteria 7).

However, besides predation, there are other explanations for the origin of the *Cloudina* holes, and these possibilities need to be considered to a better comprehension of these structures. While predation was proposed, others authors also postulated some alternative hypothesis: dissolution of microdolomite crystals, decomposition by microorganisms, microborings, and parasitism (Debrenne & Zhuravlev, 1997; Zhuravlev *et al*., 2012).

Even if the phosphatized specimens of the Dengying Formation suffered dissolution processes for preparation of the fossil material, both Brazillian and Namibian material are calcified, and an origin by dissolution of microdolomite crystal can be ruled out. Additionally, the holes in the fossils illustrated by Zhuravlev *et al*. (2012) interpreted to be formed by dissolution of microdolomite, are usually of a quadrangular outline. This shape is not observed in neither of the other occurrences (Bengtson and Zhao, 1992; Brain, 2001; Hua *et al*., 2003; this study). Thus, while it is possible that processes of dissolution created the patterns observed by Zhuravlev *et al*. (2012), the origin of the holes in Tamengo, Dengying and Nama units may rely on another cause.

Zhuravlev *et al*. (2012) also suggested that microbial decomposition was a possible hypothesis for the origin of the holes. In fact, Hof and Briggs (1992) observed circular pits on crustaceans cuticle caused by the activity of bacteria. But these circular holes had a highly variable size (<10 μm e ≥ 100 μm, based on Fig 2 of Hof and Briggs, 1992) and high density and proximity of the holes (Hof & Briggs, 1992: Fig 2A). This situation was not seen in the holes of *Cloudina*, since they commonly occur as single and isolated holes.

Although there are parameters to identify a predatory boring hole, it is not always possible to satisfactorily distinguish between holes caused by predators from that produced by ectoparasites (Baumiller *et al*., 1999; Kowalewski *et al*., 2000, Kowalewski, 2002; Harper, 2003). Nevertheless, there are some lines of evidence that can indicate parasitic origin for circular pits, such as fixations scars and various perforations in the same individual (Matsukuma, 1978; Baumiller, 1990; Kowalewski, 2002). Both situations were not found in perforated *Cloudina* from Brazil, China or Namibia.

Supplementary Figures

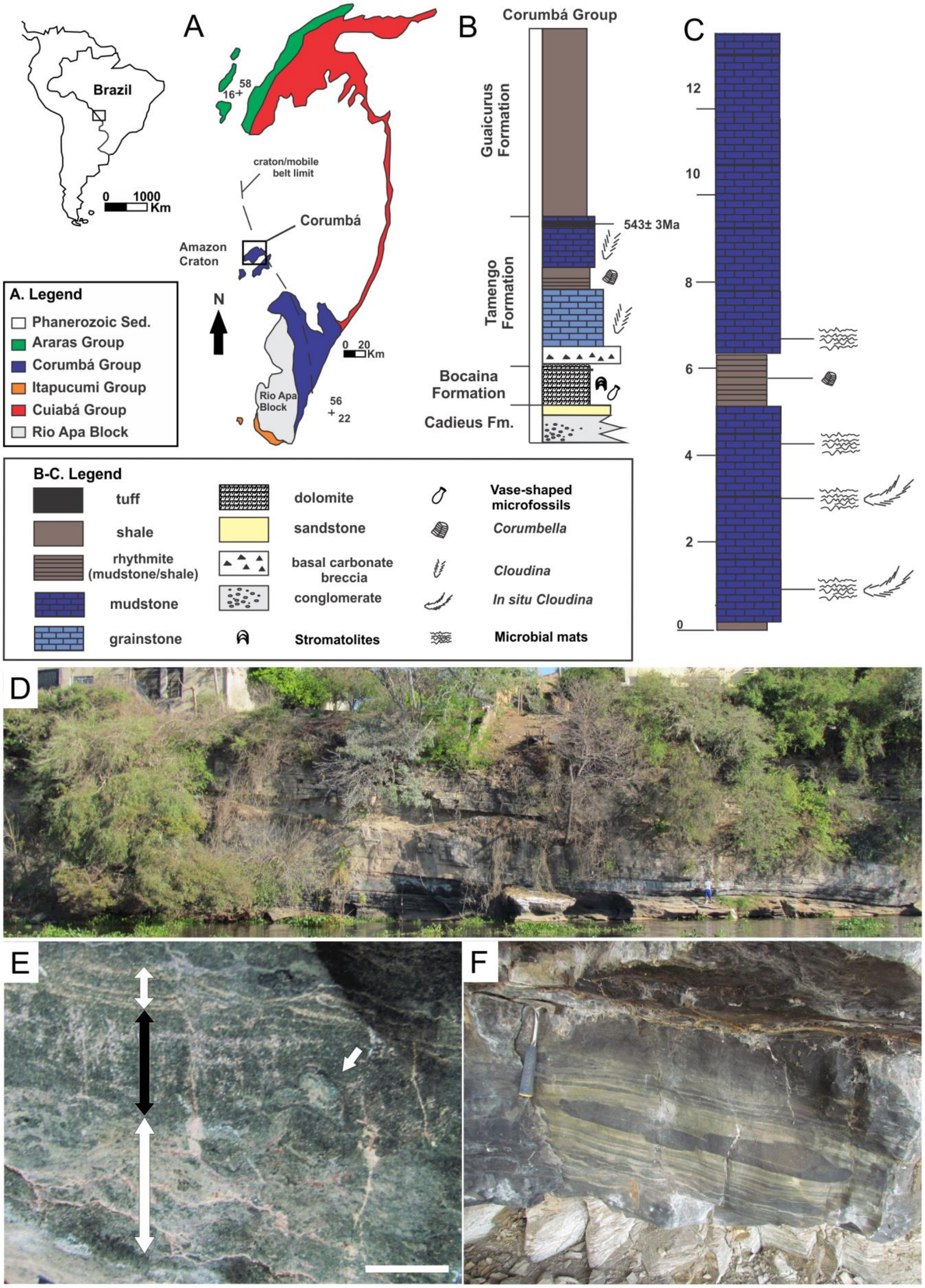
A
Brazil
0 1000 Km
58
16
craton/mobile belt limit
Corumbá
Amazon Craton
N
0 20 Km
Rio Apa Block
56
22
A. Legend
Phanerozoic Sed.
Araras Group
Corumbá Group
Itapucumi Group
Cuiabá Group
Rio Apa Block
B
Corumbá Group
Guaicurus Formation
543± 3Ma
Tamengo Formation
Bocaina Formation
Cadieus Fm.
C
0
2
4
6
8
10
12
B-C. Legend
tuff
shale
rhythmite (mudstone/shale)
mudstone
grainstone
dolomite
sandstone
basal carbonate breccia
conglomerate
Stromatolites
Vase-shaped microfossils
Corumbella
Cloudina
In situ Cloudina
Microbial mats
D
E
F

Supplementary Figure S1. Geological context of Tamengo Formation. (**A**) Geological map showing the unities associated with the Paraguay Belt, the rectangle indicating the location of sampling area. Modified from Boggiani *et al*. (2010). (**B**) General stratigraphic framework of Corumbá Group and its formations. (**C**) Stratigraphic column of the Ladário outcrop with the stratigraphic position of microbial mat textures and *in situ Cloudina*. (**D**) Field outcrop of the Ladário section studied here (19° 0' 2.89" S- 57° 36' 25.52 W). (**E**) Field photography of *in situ Cloudina* associated with two levels of crinkly laminae (white double arrows) and mudstone (black double arrows); scale bar: 1 cm. (**F**) Field photography of crinkly laminae; stratigraphic hammer for scale.

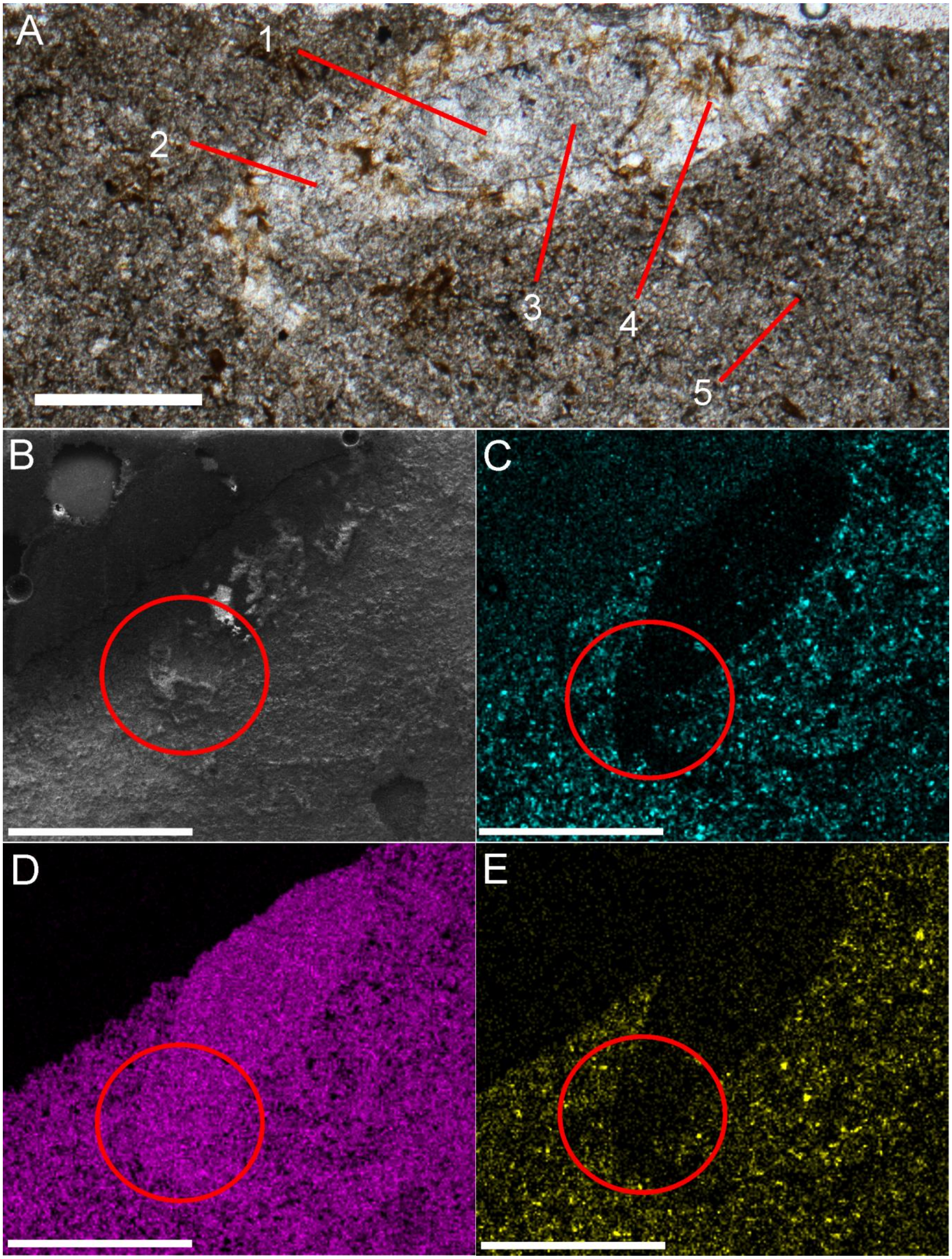

Supplementary Figure S2. Thin section and EDS mapping of autochthonous *Cloudina* with calcite cements. (**A**) Oblique section of an autochthonous specimen of *Cloudina* with

textural characteristics indicated by the numbers: 1) sparry calcite inside the central canal; 2) Microspar cements inside the flanges; 3) microspar filling the central canal; 4) sparry calcite with intercrystalline goethite; 5) microspar and siliciclastic components of the host rock framework. (**B**) SEM image of the specimen in (A) that was analyzed by EDS mapping. (**C**) Map of silicon. (**D**) Map of calcium. (**E**) Map of aluminum. Scale bars (**A–C**, **E**) 2 mm, (**D**, **F**) 1 mm.

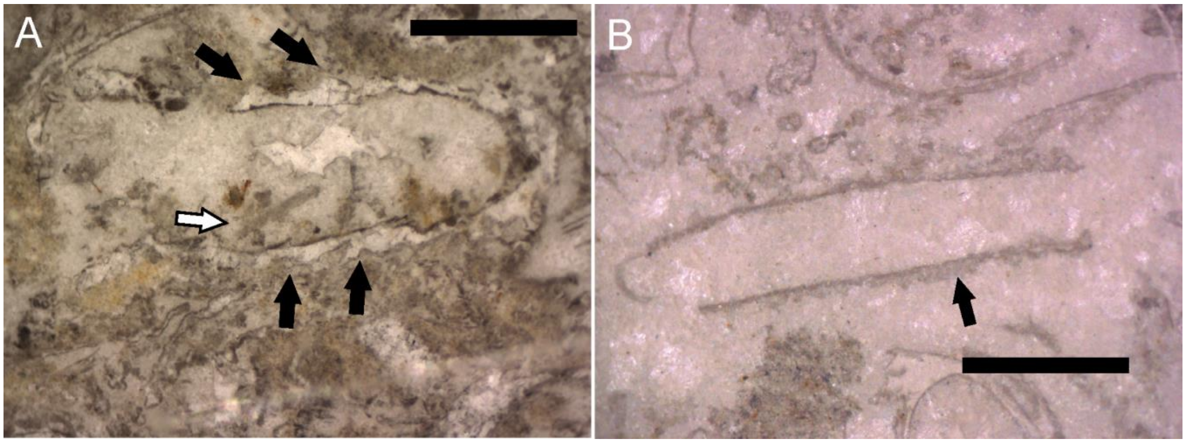

Supplementary Figure S3. Calcite cements in transported *Cloudina* shells (**A**) Early marine calcite cements providing a ghost of the funnel-in-funnel structure (black arrows), while the central canal is filled with sediments (white arrow) and flanges abraded by transport. (**B**) Early marine calcite cements covering the external surface of the tube (black arrow), but not in the interior of the tube. Scale bar, 1 mm.

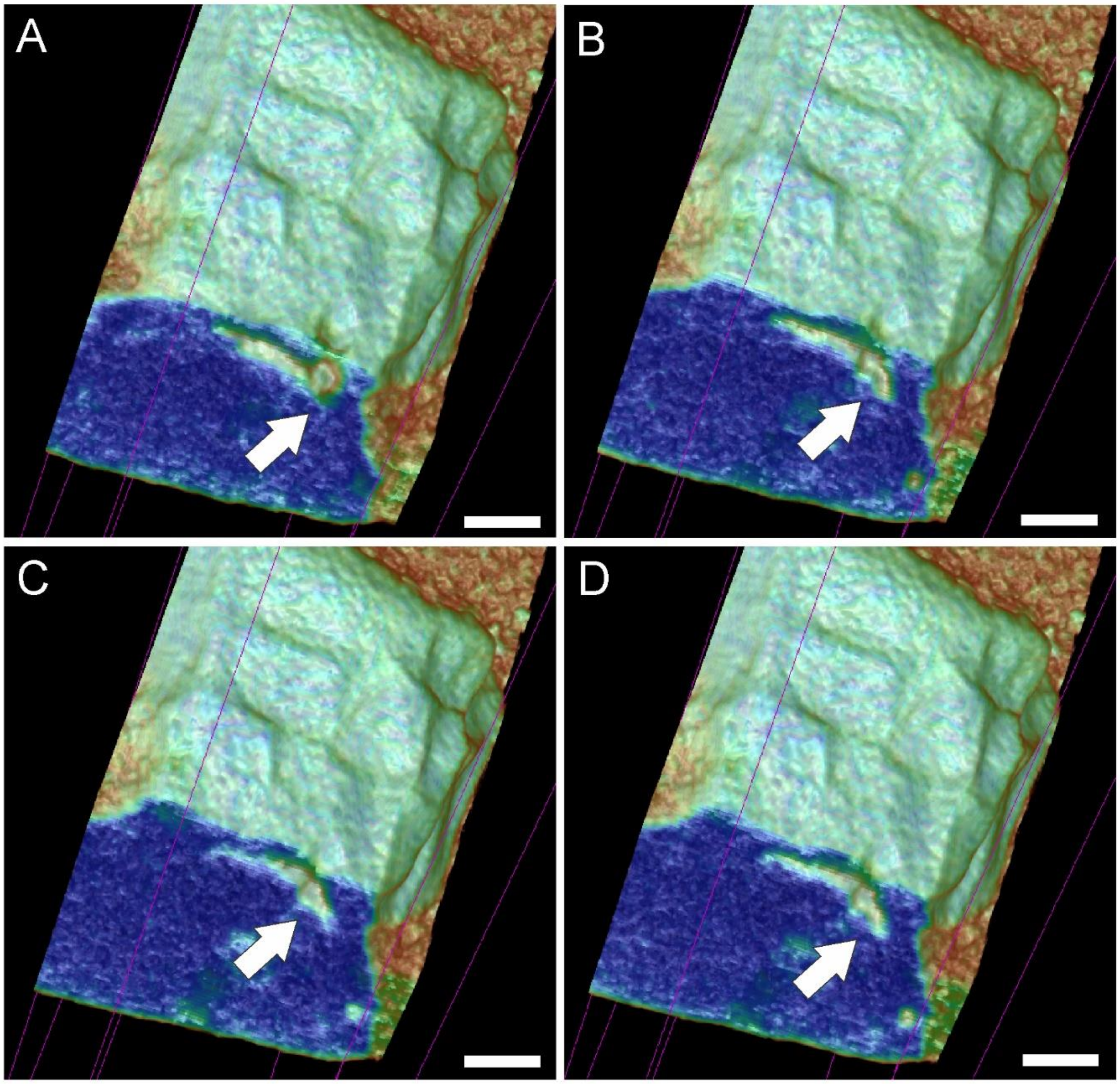

Supplementary Figure S4. Micro-CT of predatory hole in *Cloudina*. (**A**–**D**) Vertical sections of microtomographic images of a *Cloudina* specimen bearing a borehole. Note the prolongation of the hole towards the interior of the tube (arrows). Scale bar, 0.4 mm.

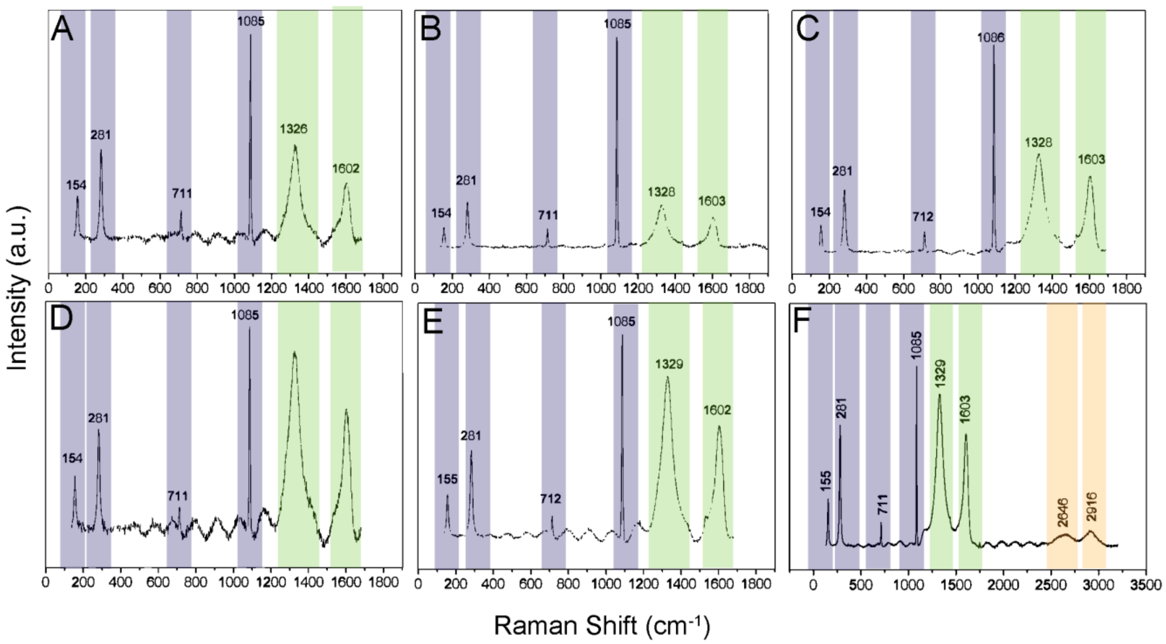

Supplementary Figure S5. (**A**–**F**) Raman point of *Cloudina* shells showing the calcite bands (in blue, with the most intense at *ca.* 1086 cm^{-1}, corresponding to the symmetric stretch of CO_3^{-2}) and primary (D-band, disordered C-C, in green) and secondary (G-band, graphitic C-C, in orange) bands of kerogen.

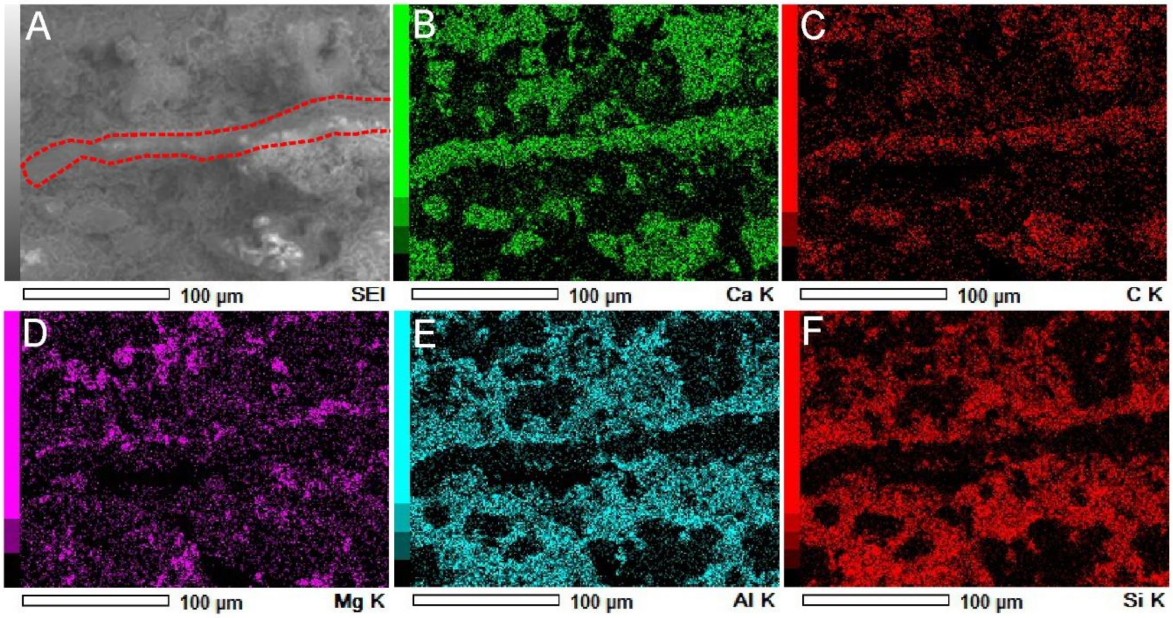

Supplementary Figure S6. EDS mapping of the *Cloudina* shell in cross-section. (**A**) Original image with the shell highlighted with the dotted red line. (**B**) Map of calcium. (**C**) Map of carbon. (**D**) Map of Mg. (**E**) Map of aluminum. (**F**) Map of silicon.

References

Almeida, F. F. M. Geologia da Serra da Bodoquena (Mato Grosso), Brasil. *Boletim da Divisão de Geologia e Mineralogia, DNPM*, **219**, 1–96 (1965).

Almeida, F. F. M. Província Tocantins, setor Sudoeste. (Almeida, F. F. M. & Hasui, Y. eds.): *O Pré-Cambriano do Brasil*. São Paulo. Edgard Blücher. 265–281 (1984).

Alvarenga, C. J. S., & Trompette, R. Evolução tectônica brasiliana da Faixa Paraguai: a estruturação da região de Cuiabá. *Revista Brasileira de Geociências*, 23(1), 18-30 (1993).

Alvarenga, C. J. S. *et al*. Paraguay and Araguaia belts. (Cordani, U. G., Milani, E., Thomaz-Filho, A., & Campos, D. A. eds.): *Tectonic Evolution of South America*. 31st International Geological Congress. Rio de Janeiro. 183–193 (2000).

Alvarenga, C. J. S. *et al*. The Amazonian Palaeocontinent. (Gaucher, C., Sial, A. N., Halverson, G. P., & Frimmel, H. E. eds.): *Neoproterozoic–Cambrian Tectonics, Global Change and Evolution: A Focus on Southwestern Gondwana*. Developments in Precambrian Geology. Elsevier. **16**, 15–28 (2009).

Amthor, J. E. *et al*. Extinction of *Cloudina* and *Namacalathus* at the Precambrian-Cambrian boundary in Oman. *Geology* **31(5)**, 431–434 (2003).

Babinski, M. *et al*. 2008. U– Pb shrimp geochronology and isotope chemostratigraphy (C, O, Sr) of the Tamengo Formation, Southern Paraguay Belt, Brazil. (Linares, E., Cabaleri, N. G., Do Campo, M. D., Ducós, E. I., Panarello, H. O. eds.): *VI South American Symposium on Isotope Geology*. Proceedings in CD-ROM, Buenos Aires. 1851–6963 (2008).

Baumiller, T. K. Non-predatory drilling of Mississippian crinoids by platyceratid gastropods. *Palaeontology* **33**, 743–748 (1990).

Baumiller, T. K., Leighton, L. R. & Thompson, D. L. Boreholes in Mississippian spiriferide brachiopods and their implications for Paleozoic gastropod drilling. *Palaeogeogr. Palaeoclimatol. Palaeoecol.* **147**, 283–289 (1999).

Bengtson, S. & Yue, Z. Predatorial borings in late Precambrian mineralized exoskeletons. *Science* **257**, 367–369 (1992).

Boggiani, P. C. Análise estratigráfica da Bacia Corumbá (Neoproterozoico)- Mato Grosso do Sul. Ph.D. Thesis, Institute of Geosciences, University of São Paulo, Brazil. (1998).

Boggiani, P. C. *et al*. Chemostratigraphy of the Tamengo Formation (Corumbá Group, Brazil): a contribution to the calibration of the Ediacaran carbon-isotope curve. *Precambrian Res.* **182**, 382–401 (2010).

Brain, C. K. Some observations on *Cloudina*, a terminal Proterozoic index fossil from Namibia. *J. Afr. Earth Sci.* **33**, 475–480 (2001).

Debrenne, F. & Zhuravlev, A. Yu. Cambrian food web: a brief review: *Geobios, Mémoire Spécial* **20**, 181–188 (1997).

Fairchild, T. R. *et al*. Evolution of Precambrian life in the Brazilian geological record. *Int. J. Astrobiol.* **11**, 309–323 (2012).

Gaucher, C. *et al*. Integrated correlation of the Vendian to Cambrian Arroyo del Soldado and Corumbá Groups (Uruguay and Brazil): palaeogeographic, paleoclimatic and palaeobiologic implications. *Precambrian Res.* **120**, 241–278 (2003).

Grotzinger, J. P. *et al*. Biostratigraphic and geochronologic constraints on early animal evolution. *Science* **270**, 598–604 (1995).

Harper, E. M. Assessing the importance of drilling predation over the Palaeozoic and Mesozoic. *Palaeogeogr. Palaeoclimatol. Palaeoecol.* **210**, 185–198 (2003).

Hof, C. H. J. & Briggs, D. E. G. Decay and mineralization of mantis shrimps (Stomatopoda: Crustacea) - A key to their fossil record. *Palaios* **12**, 420–438 (1997).

Hua, H., Pratt, B. R. & Zhang, L.-Y. Borings in *Cloudina* shells: complex predator-prey dynamics in the terminal Neoproterozoic. *Palaios* **18**, 454–459 (2003).

Kowalewski, M. The fossil record of predation: an overview of analytical methods. *Paleontological Society Papers* **8**, 3–42 (2002).

Kowalewski, M. *et al*. Drill holes in shells of Permian benthic invertebrates. *J. Paleo. 74*, 532–543 (2000).

Matsukuma, A. Fossil boreholes made by shell-boring predators or commensals, Part I: Boreholes of capulid gastropods. *Venus* **37**, 29–45 (1978).

Pacheco, M. L. A. F., Leme, J. M. & Machado, A. F. Taphonomic analysis and geometric modeling for the reconstitution of the Ediacaran metazoan *Corumbella werneri* Hahn *et al*. 1982 (Tamengo Formation, Corumbá Basin, Brazil). *Journal of Taphonomy* **9**, 269–283 (2011).

Spangenberg, J. E. *et al*. Redox variations and bioproductivity in the Ediacaran: evidence from inorganic and organic geochemistry of the Corumbá Group, Brazil. *Gond. Res.* **26**, 1186–1207 (2013).

Trompette, R., Alvarenga, C. J. S. & Walde, D. Geological evolution of the Neoproterozoic Corumba graben system (Brazil). Depositional context of the stratified Fe and Mn ores of the Jacadigo Group. *J. South Am. Earth Sci.* **11**, 587–597 (1998).

Zaine, M. F. & Fairchild, T. R. Comparision of *Aulophycus lucianoi* Beurlen & Sommer from Ladário (MS) and the genus *Cloudina* Germs, Ediacaran of Namibia. *Anais da Academia Brasileira de Ciências* **57**, 130 (1985).

Zaine, M. F. & Fairchild, T. R. Novas considerações sobre os fósseis da Formação Tamengo, Grupo Corumbá, SW do Brasil. Anais do X Congresso Brasileiro de Paleontologia. Rio de Janeiro. *Resumo das Comunicações* **2**, 797–807 (1987).

Zaine, M. F. Análise dos fósseis de parte da Faixa Paraguai (MS, MT) e seu contexto temporal e paleoambiental. Ph.D. Thesis, Institute of Geosciences, University of São Paulo, 215 p (1991).

Zhuravlev, A. Y. *et al*. New Finds of Skeletal Fossils in the Terminal Neoproterozoic of the Siberian Platform and Spain. *Acta Palaeontol. Pol.* **57(1)**, 205–224 (2012).
